# Glossopharyngeal Neuralgia Secondary to COVID-19: A Case Report

**DOI:** 10.7759/cureus.26817

**Published:** 2022-07-13

**Authors:** Bao Q Nguyen, Darrick J Alaimo

**Affiliations:** 1 Neurology, Darrick J. Alaimo, MD, LLC, Rochester, USA

**Keywords:** coronavirus disease 2019, covid-19, pain management, glossopharyngeal nerve, world pandemic, glossopharyngeal neuralgia

## Abstract

Glossopharyngeal neuralgia (GPN) is a painful condition characterized by stabbing pain throughout the glossopharyngeal nerve distribution. Since the beginning of the coronavirus disease 2019 (COVID-19) pandemic, we have learned that COVID-19 may induce neurological symptoms and complications. This case report presents a 54-year-old patient diagnosed with GPN, potentially secondary to COVID-19. The pain resolved spontaneously in three months without the need for medication. We discuss our diagnostic approach for this patient and propose a possible theory about the relation between cranial neuralgias and COVID-19.

## Introduction

Glossopharyngeal neuralgia (GPN) is a rare syndrome distinguished by distinct paroxysmal pain along the distribution of the ninth cranial nerve (CN IX), also known as the glossopharyngeal nerve. GPN only accounts for 0.2-1.3% of all types of cranial neuralgias. One study of the population in Rochester, Minnesota, from 1945 to 1984, showed a crude incidence rate of 0.7 per 100,000 population. The risk increases with age in males and females, reaching a peak at the age of 75 years [1].

Since the WHO declared the coronavirus disease 2019 (COVID-19) a global pandemic on March 11, 2020, researchers worldwide had conducted many studies about severe acute respiratory syndrome coronavirus 2 (SARS-CoV-2). Neurological symptoms and complications associated with the virus have been reported [2], such as gustatory dysfunction, headache, olfactory dysfunction, Guillain-Barré syndrome, encephalitis, and meningitis. Although cranial nerve (CN) involvement was previously reported [3], we could hardly find any systemic literature review of cranial neuralgias related to COVID-19.

Hereby, we report a 54-year-old man who presented with pathognomonic symptoms of GPN in the course of COVID-19. After ruling out all the possible known etiologies of GPN, we diagnosed him with GPN potentially secondary to COVID-19. The pain entirely resolved after three months without the need for medications. Although his GPN and COVID-19 might happen by chance, we think this virus could cause cranial neuralgias considering its well-established neuroinvasive characteristics.

To the best of our knowledge, this is the first case in which GPN developed as part of COVID-19's clinical manifestations. We hope our report will contribute to understanding SARS-CoV-2 and encourage more extensive studies in the future.

## Case presentation

A 54-year-old right-handed Caucasian man presented to our clinic with pain in the left ear and pharynx when swallowing. He was in his usual state of health until three months ago when he started feeling pain in his left ear that he thought was just an ear infection. He developed fever, chills, sweats, and fatigue a few days later. A polymerase chain reaction (PCR) test confirmed he had a COVID-19 infection. The fever, chills, sweats, and fatigue lasted only for several days, but the pain continued. When he swallowed, he would have a sharp shooting pain starting at the left tonsil, radiating upwards to the pharynx, and ending inside his left ear canal. He had approximately one episode for each swallow, which lasted for about one second and then entirely resolved. "Gently swallowing" and drinking ice water did alleviate the pain. It became worse with "big" gulps, coughing, or sneezing. Maximum pain intensity was 10/10, with 10 being the worst pain of life. He had never developed any numbness, tingling, weakness, or dysphagia. He has not noticed any change in his usual level of tinnitus and hearing loss or voice.

A dentist evaluated and suggested that bruxism or tonsillitis might cause the pain. As part of treatment, he had a throat culture and started taking clindamycin, oral corticosteroid, and ibuprofen, which were ineffective. After the throat culture returned negative, he stopped all the medications.

An otorhinolaryngologist suspected he might have GPN and ordered a magnetic resonance imaging (MRI) of the soft tissue of the neck with and without contrast, which was unrevealing. He referred the patient to our clinic afterward.

Past medical history was unremarkable except for snoring at night. On physical examination, he was overweight with a BMI of 26.3, well-developed, and well-nourished with stable vital signs. Head, eyes, ears, nose, and throat exam showed normocephalic, no oropharyngeal lesions, and normal tonsils bilaterally. Tympanic membranes were intact on the left but poorly visualized on the right due to cerumen. He had no change in pain symptoms with palpation or movement of the pinna or tragus pressure bilaterally. There was no tenderness to palpation at the temporomandibular joints.

On neurologic examination, the patient was alert and oriented. His speech was fluent without dysarthria or aphasia. Attention, concentration, memory, and fund of knowledge were intact. The visual fields were full. There was no papilledema, hemorrhage, or exudate on the funduscopic examination. The pupils were equal, round, and reactive to light. Extraocular movements were intact without nystagmus. The facial sensation and the facial strength, including cheek puff, eye closure, and frontalis muscle strength, were normal. Gag reflex was normal bilaterally. The palate elevated symmetrically, and the uvula was at the midline. Tongue protrusion and jaw closure were normal. The hearing was intact to finger rub bilaterally. Sternocleidomastoid and trapezius strength was normal. The patient had usual muscle bulk, tone, and strength in bilateral upper and lower extremities. Sensations, coordination, and gait were normal. Deep tendon reflexes and plantar responses were normal and symmetric in the arms and legs.

Given the clinical course and the nature of his pain, we diagnosed him with GPN. After ruling out possible structural lesions in the brainstem from his previous neck MRI, we diagnosed him with GPN potentially secondary to COVID-19. We recommended oral neuropathic pain medication such as gabapentin, pregabalin, or oxcarbazepine and topical anesthetic agents as adjuvants. He decided to take topical anesthetic agents only and might consider oral medications if the symptoms worsened in the upcoming days. We discussed with him that we might consider a brain MRI if his symptoms showed no improvement in the subsequent follow-up.

At the follow-up visit three months later, he reported that he had never tried the topical anesthetic agents, but the pain had entirely resolved.

## Discussion

Patients with GPN typically present with paroxysmal, severe, stabbing pain involving the ear, tonsillar fossa, the base of the tongue, or beneath the jaw angle [4]. The pain radiates from the oropharynx upward to the ear. The duration of attacks lasts seconds to minutes, but they can leave a low-grade, constant, dull background pain afterward. Patients can experience many dozen attacks a day. Exacerbating factors include chewing, swallowing, coughing, speaking, yawning, specific tastes, or touching the neck or external auditory canal. The examination is usually benign, emphasizing the critical role of the history-taking process. Any infection or compression along the glossopharyngeal nerve pathway from the brainstem to the end organs leads to hyperexcitability of the nerve and produces such pain.

Due to the rarity of GPN and its close anatomical relation to the oropharyngeal region, patients with GPN are usually misdiagnosed or even undergo unnecessary diagnostic studies [5]. Our patient was initially diagnosed with bruxism and tonsillitis and was prescribed an ineffective medication. Fortunately, the otorhinolaryngologist ordered an MRI of the soft tissue of the neck with and without contrast capturing good views of the brainstem. The unremarkable result helped us rule out structural lesions associated with GPN (e.g., mass lesion and vascular pathology) [6]. The neuralgic pain started when the patient was having COVID-19; therefore, we suspected that SARS-CoV-2 might be our patient's most likely cause of GPN. If the pain got worse, which fortunately did not happen in our patient, we would consider ordering an MRI of the brain to investigate the cause further.

Since the COVID-19 pandemic, the most common known neurological symptoms and complications include encephalopathy, acute cerebrovascular diseases, and acute polyradiculopathy or neuropathies [7,8]. The literature review suggested four possible neuropathogenesis of SARS-CoV-2: systemic hypoxemia [9], indirect invasion of the virus through angiotensin-converting enzyme 2 [10-12], immune dysfunction [13,14], and the direct viral invasion of the nervous system [15,16]. As our patient only experienced a mild course of infection, it was unlikely that he underwent a hypoxemic crisis or immune dysfunction. Therefore, we thought his condition was due to the direct or indirect attacks of the virus against CN IX.

Cranial nerve dysfunction is commonly found in patients with COVID-19 infection [3]. The olfactory nerve (the first cranial nerve, CN I) is considered the most vulnerable target of SARS-CoV-2. In the meta-analysis of 83 studies involving more than 27,000 patients, 48% of participants reported experiencing olfactory dysfunction [17]. Meanwhile, involvement of other cranial nerves, CN IX as in our patient, seems less prevalent. Doblan et al. proposed that since SARS-CoV-2 primarily attacks the respiratory tract, the extent of a cranial nerve innervating the respiratory system may correlate with its vulnerability to the virus [3]. In patients with symptoms of cranial nerve damage, we recommend paying attention to all cranial nerves anatomically related to the respiratory tree, including CN I, CN V (trigeminal nerve), CN VII (facial nerve), CN IX, and CN X (vagus nerve), since the virus may already invade the CNS at this stage of infection.

Considering the extreme excruciating GPN pain, pain control should be prioritized to improve quality of life. Similar to treatment for trigeminal neuralgia (TN), carbamazepine and oxcarbazepine are the first-line medications for GPN. Despite not taking oxcarbazepine and anesthetic agents, our patient tolerated the symptoms and was eventually pain-free after three months. A literature review discovered that three patients manifested TN during their mild COVID-19 course [18,19]. Two patients in a case series by Bohania et al. were asymptomatic in one to two weeks with carbamazepine [18], while Molina-Gil et al. reported that their patient's TN resolved completely as his COVID-19 symptoms got improved [19]. Contradicting CN I's long-term and even permanent damage [20], SARS-CoV-2-induced neuralgias seem to be temporary. There is a chance that our patient only had GPN coincidently during his COVID-19, and those conditions might not be related. However, until further investigations are conducted, we suggest that healthcare providers inform their patients of this potentially good prognosis to calm their anxiety.

## Conclusions

We believe that COVID-19 might have been one of the secondary causes of GPN. MRI of the brainstem is a must to rule out structural lesions. Healthcare providers should examine the possible damage to every cranial nerve innervating the respiratory tract in patients with COVID-19-related cranial nerve dysfunction. GPN, probably caused by SARS-CoV-2, fully resolved without medical treatment in our case. The first-line medications include carbamazepine or oxcarbazepine, which can be combined with topical anesthetic agents.
